# Daurinol Attenuates Autoimmune Arthritis via Stabilization of Nrp1–PTEN–Foxp3 Signaling in Regulatory T Cells

**DOI:** 10.3389/fimmu.2019.01526

**Published:** 2019-07-17

**Authors:** Min-Jung Park, Su-Jin Moon, Eun-Jung Lee, Eun-Kyung Kim, Jin-Ah Baek, Se-Young Kim, Kyung Ah Jung, Seung Hoon Lee, Jeong Won Choi, Da-Som Kim, Jun-Ki Min, Sung-Hwan Park, Dongyun Shin, Mi-La Cho

**Affiliations:** ^1^The Rheumatism Research Center, Catholic Research Institute of Medical Science, College of Medicine, The Catholic University of Korea, Seoul, South Korea; ^2^Division of Rheumatology, Department of Internal Medicine, Uijeongbu St. Mary's Hospital, College of Medicine, The Catholic University of Korea, Seoul, South Korea; ^3^College of Pharmacy, Gachon University, Incheon, South Korea

**Keywords:** daurinol, regulatory T cells, stability, FOXP3 hypomethylation, rheumatoid arthritis, neuropilin 1

## Abstract

Optimizing Treg function and improving Treg stability are attractive treatment strategies for treating autoimmune rheumatoid arthritis (RA). However, the limited number of circulating Tregs and questions about the functional stability of *in vitro*-expanded Tregs are potential limitations of Treg-based cell therapy. The aim of this study was to analyze the regulatory effect of daurinol, a catalytic inhibitor of topoisomerase IIα, on Th cell differentiation and to evaluate their therapeutic potential in a preclinical experimental model of RA. We investigated the effect of daurinol on T cell differentiation by flow cytometry. Foxp3 stability and methylation were analyzed by suppression assays and bisulfite pyrosequencing. Daurinol was treated in the collagen-induced arthritis (CIA) model, and the effects *in vivo* were determined. We found that daurinol can promote Treg differentiation and reciprocally inhibit Th17 differentiation. This Treg-inducing property of daurinol was associated with decreased activity of Akt–mTOR and reciprocally increased activity of neuropilin-1 (Nrp1)–PTEN. Daurinol treatment inhibited aerobic glycolysis in Th17 conditions, indicating the metabolic changes by daurinol. We found that the daurinol increase the Treg stability was achieved by Foxp3 hypomethylation. *In vivo* daurinol treatment in CIA mice reduced the clinical arthritis severity and histological inflammation. The Treg population frequency increased and the Th17 cells decreased in the spleens of arthritis mice treated with daurinol. These results showed the anti-arthritic and immunoregulating properties of daurinol is achieved by increased differentiation and stabilization of Tregs. Our study provides first evidence for daurinol as a treatment for RA.

## Introduction

Rheumatoid arthritis (RA) is a chronic, systemic, inflammatory disease characterized by tumor-like growth of the synovium and infiltration of immune cells through the affected joints. Interleukin 17 (IL-17)-secreting CD4+ T cells (Th17) have been shown to play a crucial role in the pathogenesis of autoimmune diseases (1). It has become clear that these proinflammatory cytokines including tumor necrosis factor α (TNF-α), IL-6, and IL-1 play together with other inflammatory mediators IL-17 in an additive or synergistic way (2). Many studies have identified the pivotal roles of IL-17 and Th17 cells in the development and progression of RA (3). In contrast to Th17 cells, regulatory T cells (Tregs) comprise an indispensable mediator that sustains immune tolerance to self-antigens and helps to maintain immune homeostasis (4). The Th17–Treg paradigm is vital to understanding the pathogenesis of T cell-mediated autoimmune disorders such as RA (5, 6), and systemic lupus erythematosus (SLE) (7).

RA patients with a high number of CD4+CD25+ Tregs in whole blood respond better to the anti-TNF agent infliximab than do those with lower number of Tregs measured at baseline before the treatment (8). This finding suggests that a strategy to expand the Treg population may be helpful in controlling RA disease activity and augmenting treatment efficacy in RA patients. In addition to the lower number of Tregs in some RA patients (9), several studies provided evidence of disturbed immunoregulatory function of these cells in RA patients (10, 11). CD4+CD25+ Tregs present in RA patients do not express *FOXP3* (10). Foxp3 acts to stabilize the immunoregulatory function of Tregs (12), and appears to be related to defects in Tregs function in RA patients. Foxp3 is required for Treg development and function.

Daurinol is a natural aryl naphthalene lactone that can be isolated from the traditional medicinal plant *Haplophyllum dauricum* (13). Our previous studies have identified its anti-cancer property (14, 15). The chemical structure of daurinol is similar to that of VP-16, which is also known as etoposide, a widely used clinical anticancer drug (16). The safety profile of daurinol differs considerably for that of etoposide. Compared with etoposide, daurinol causes little loss of body weight and less bone marrow suppression (14). Previous studies showing the therapeutic potential of daurinol have focused mainly on its ability to inhibit cell proliferation and the underlying mechanisms. Until now, no one, including our research team, has studied the anti-inflammatory or anti-arthritic efficacy of daruinol.

The aim of the present study was to examine the therapeutic potential of daurinol in RA and the underlying mechanisms, especially on modulation of T cell subsets. This is the first report of the reciprocal regulation of Th17 and Tregs by daurinol treatment *in vitro* and *in vivo*. We found that daurinol induced the proliferation and differentiation of Tregs from naïve CD4+ T cells and that the increase in immunoregulatory function and Treg number induced by daurinol was achieved through Foxp3 induction via Foxp3 hypomethylation. We suggest that daurinol has potential as a novel compound to inhibit RA through a Treg-specific mechanism involving expansion and stabilization of this population.

## Materials and Methods

### Mice

DBA/1J and C57BL/6 (B6) mice, 8–10 weeks of age, were purchased from OrientBio (Sungnam, Korea) and were maintained under specific-pathogen-free conditions in an animal facility. The protocols used in this study were approved by the Animal Care and Use Committee of the Catholic University of Korea.

### Induction of Arthritis and Daurinol Treatment

Collagen-induced arthritis (CIA) was induced in DBA1/J mice (each group: *n* = 10). Mice were immunized with 100 μg of chicken CII (Chondrex Inc., Redmond, WA, USA) dissolved overnight in 0.1 N acetic acid (4 mg/ml) in complete Freund's adjuvant or incomplete Freund's adjuvant (Chondrex Inc.). The immunization was performed intradermally into the base of the tail. The mice were randomly assigned to three experimental groups (*n* = 10) and treated with daurinol (5 mg/kg or 25 mg/kg of body weight) or vehicle by oral gavage three times a weeks for 3 weeks since 3 weeks after 1st CII immunization.

### Clinical Scoring and Histological Assessment of Arthritis

The onset and severity of arthritis were measured visually twice per week based on the appearance of arthritis in the joints, based on the previously described scoring system (17). Detailed experimental procedures are described in Supplementary section Materials and Methods.

### Measurement of Cytokine and IgG Levels

The concentrations of IFN-γ, and IL-17 in culture supernatants and serum were measured using a sandwich enzyme-linked immunosorbent assay (ELISA Duoset; R&D Systems, Lille, France). Serum levels of IgG, IgG1, and IgG2a antibodies were measured using a commercially available ELISA kit (Bethyl Laboratories).

### Murine and Human T Cell Isolation and Differentiation

To purify mouse splenic or human CD4^+^ T cells, the splenocytes were incubated with CD4-coated magnetic beads and isolated using magnetic-activated cell sorting separation columns (Miltenyi Biotec, Bergisch Gladbach, Germany). Mouse Th17 cell differentiation was induced by treatment with anti-CD3 (0.5 μg/ml); and soluble anti-CD28 (0.5 μg/ml), IL-6 (20 ng/ml) and TGF-β (2 ng/ml), anti-IFN-γ, and anti-IL-4 antibodies (each at a concentration of 5 μg/ml). HumanTh17 cells were stimulated with plate-bound anti-CD3 (0.5 μg/ml); and soluble anti-CD28 (0.5 μg/ml), anti-IFN-γ (2 μg/ml), anti-IL-4 (2 μg/ml), anti-IL-1β (20 ng/ml), and anti-IL-6 (20 ng/ml) for 72 h.

### Metabolic Assays

The ECAR were measured with an XF96 analyzer (Seahorse Bioscience). Cultured CD4 T cells were seeded at a density of 5 × 10^5^ cells per well of a XF96 cell culture microplate. Before assay, cells were equilibrated for 1 h in unbuffered XF assay medium supplemented with 0.1% Insulin-Transferrin-Selenium-Sodium Pyruvate (ITSA). Compounds were injected during the assay at the following final concentrations: 2 μM Oligomycin, 3 μM FCCP, and 5 μM Rotenone-Antimycin A.

### Crisper Cas9 Transfection

The Alt-R CRISPR/Cas9 system was carried out as previously described (18, 19). Nrp-1 or PTEN CRISPR/Cas9 vector was transfected using an Amaxa 4D-nucleofector X unit according to the manufacturer's recommendations with program DN-100 (Lonza, Cologne, Germany).

### Treg Stability

To test of Treg stability, mouse CD4+T cells were stimulated with Treg differentiation condition (CD3 and CD28 and rhTGF-b (5 ng/ml) for 3 days and restimulated with Th17 cell differentiation condition(anti-CD3 (0.5 μg/ml); and soluble anti-CD28 (0.5 μg/ml), IL-6 (20 ng/ml) and TGF-β (2 ng/ml), anti-IFN-γ, and anti-IL-4 antibodies (each at a concentration of 5 μg/ml) with or without dauirinol. After 3 days, the cell surface was stained with CD4, CD25, and FoxP3 antibodies.

### Proliferation Assay

For proliferation analysis, cells were pulsed with 1 Ci 3H-thymidine (GE Healthcare) per well for the final 8 h of the 72-h culture period. Finally, 3H-thymidine incorporation was determined using a liquid beta-scintillation counter (Beckman).

### CpG Methylation Analysis

CpG methylation analysis was determined by pyrosequencing of bisulphite-modified genomic DNA from non-treated CD4+T cells or daurinol-treated CD4+T cells. Pyrosequencing was performed using the PyroMark Q96 ID (Qiagen) machine, and results were analyzed with PyroMark CpG Software 1.0 (Qiagen). Methylation analysis was conducted by Genomictree, Daejeon, South Korea.

### Real-Time Polymerase Chain Reaction (PCR)

Messenger RNA (mRNA) was extracted using the TRI Reagent (Molecular Research Center, Inc. Cincinnati, OH, USA) according to the manufacturer's instructions. Complementary DNA was synthesized using a SuperScript Reverse Transcription system (Takara Bio Inc., Otsu, Japan). A LightCycler 2.0 instrument (software version 4.0; Roche Diagnostics, Mannheim, Germany) was used for PCR amplification. All reactions were performed using the LightCycler FastStart DNA Master SYBR Green I mix (Takara Bio Inc.), following the manufacturer's instructions. Primer sequences are described in Tables S1, S2. All mRNA levels were normalized to that of β-actin.

### Western Blotting

Proteins were separated by sodium dodecyl sulfate polyacrylamide gel electrophoresis (SDS–PAGE) and transferred to nitrocellulose membranes (Amersham Pharmacia Biotech, Buckinghamshire, UK). Membranes were stained with primary antibodies against phosphorylated (active) form of pSTAT3 (Tyr^705^, Ser^727^), STAT3, STAT5, pSTAT5, PTEN, pPTEN, Akt, pAkt (Ser^473^, Tyr^308^), pmTOR, Nrp1 (all from Cell Signaling, Danvers, MA, USA), and β-actin. A horseradish peroxidase (HRP)-conjugated secondary antibody was then added. Primary antibodies used for western blot are described in Table S3.

### Flow Cytometry

Mononuclear cells were immunostained with various combinations of the following fluorescence-conjugated antibodies: CD25, CD4, FoxP3, IL-17, CTLA-4, and glucocorticoid-induced tumor necrosis factor receptor (GITR), ICOS, C103, and PD-1. These cells were also intracellularly stained with the following antibodies: CTLA-4 (BD Biosciences), Nrp-1(R&D), IL-17, and FoxP3 (eBioscience). Prior to intracellular staining, cells were restimulated for 4 h with phorbol myristate acetate (25 ng/ml) and ionomycin (250 ng/ml) in the presence of GolgiSTOP (BD Biosciences). Intracellular staining was conducted using a kit (eBioscience), following the manufacturer's protocol. Flow cytometry was performed using a FACSCalibur instrument (BD Biosciences).

### Labeling With 5,6-Carboxyfluorescein Succinimidyl Ester (CFSE)

Mononuclear cells isolated from mice spleens were washed once in 0.1% bovine serum albumin (BSA) in PBS and labeled with 1 _l of 5 mM CFSE (Invitrogen) at a density of 10^7^ cells/ml in 0.1% BSA in PBS for 10 min at 37°C in the dark. CFSE-labeled cells were stimulated with Th17 differentiation condition treated with either vehicle and daurinol for 3 days. Flow cytometry was used to assess CFSE fluorescence.

### Confocal Microscopy and Immunostaining

Spleen tissues were obtained 8 or 14 days after BMT, snap-frozen in liquid nitrogen, and stored at −80°C. Tissue cryosections (7 μm thick) were fixed in 4% (v/v) paraformaldehyde and stained using fluorescein isothiocyanate (FITC)-, phycoerythrin (PE)-, PerCP-Cy5.5-, or allophycocyanin -conjugated monoclonal antibodies to mouse CD4, CD25, pSTAT3 (Ser^727^), IL-17, Foxp3 (eBioscience), and Nrp1 (R&D). After incubation overnight at 4°C, stained sections were visualized by confocal microscopy (LSM 510 Meta; Zeiss, Göttingen, Germany).

### Immunohistochemistry

Immunohistochemistry was performed using the VECTASTAIN ABC kit (Vector Laboratories, Burlingame, CA, USA). Tissues were first incubated with the primary anti-IL-17, anti-Foxp3, anti-TNF-a, anti-IL-1β, anti-IL-6, anti-RANK, and anti-RANKL antibodies overnight at 48°C. The primary antibody was detected with a biotinylated secondary linking antibody, followed by incubation with a streptavidin–peroxidase complex for 1 h. The final color product was developed using DAB chromogen (DAKO, Carpinteria, CA, USA).

### Microarray Data

T cells were isolated from WT mice, and treated with Th17 condition with or without daurinol. Affymetrix microarrays HT_MG-430A were used to measure the resulting mRNA. Expression data was preprocessed using the RMA algorithm followed by quantile normalization. To identify differentially expressed genes of interest, real-time PCR (RT-PCR) was used to validate the microarray results.

### Statistical Analysis

Data are presented as the mean ± standard deviation (s.d.). The Mann–Whitney *U* test or Student *t*-test was used for comparing values between two groups. One-way analysis of variance followed by Bonferroni's *post-hoc* test was used to compare the differences between three or more groups. To assess the Gaussian distribution and the equality of variance, the Shapiro–Wilk test and Levene test were used, respectively. Differences between arthritis incidences at a given time point were analyzed by the χ2 contingency analysis. The program used for the statistical analysis was the SPSS statistical software package, standard version 16.0 (SPSS, Chicago, IL, USA). *P*-values < 0.05 (two-tailed) were considered significant.

## Results

### Reciprocal Regulation of Th17 and Tregs by Daurinol Treatment

**Figure 1:**
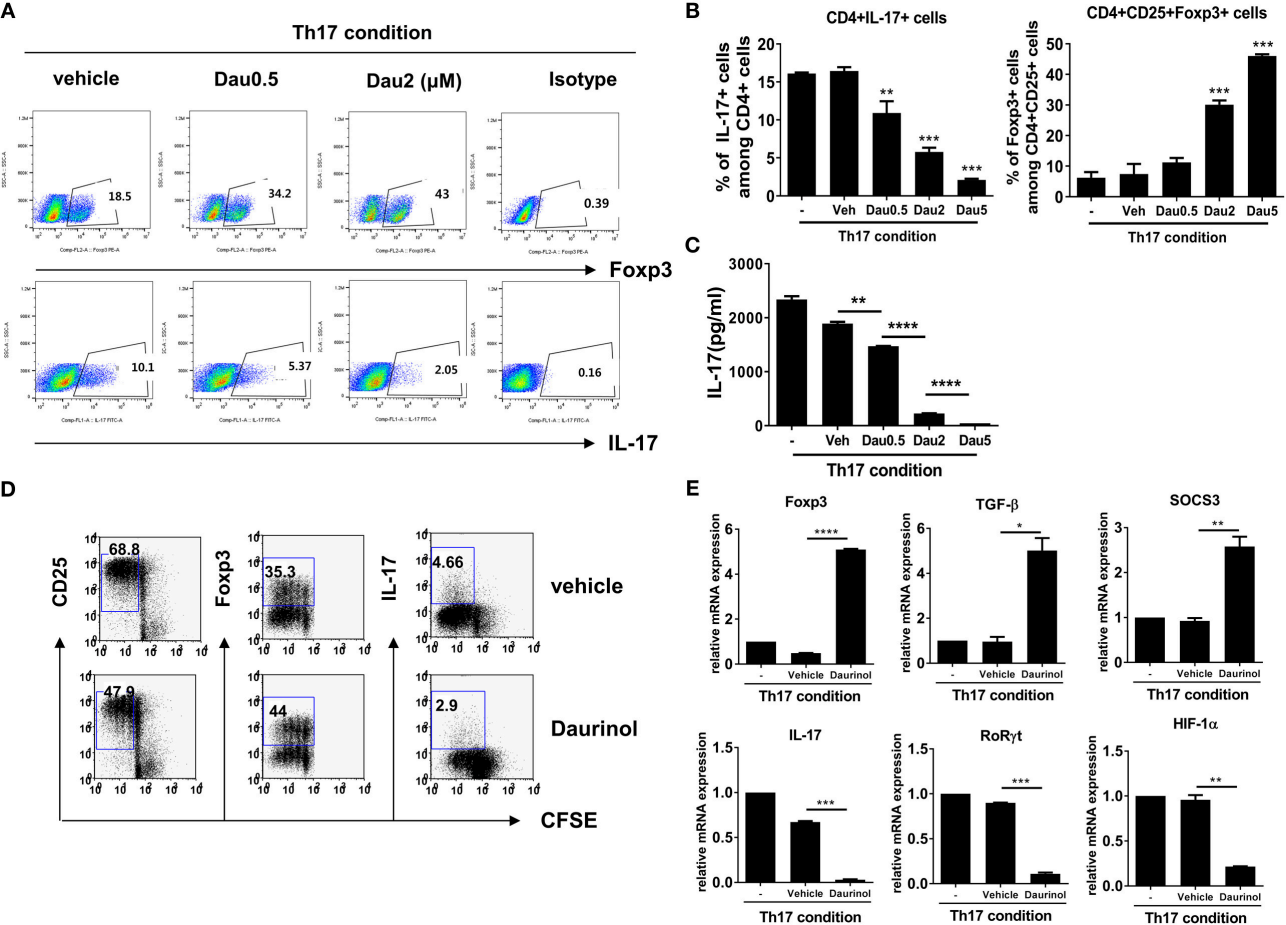
Reciprocal effects of daurinol on Th17 and Treg cell differentiation in murine CD4+ T cells. Splenic CD4+ T cells from DBA/1J mice were cultured under the Th17-inducing condition (cultured with plate-bound anti-CD3 (0.5 μg/ml), soluble anti-CD28 (0.5 μg/ml), anti-interferon-γ anti-IFNγ; [5 μg/ml **(A)** or 2 μg/ml **(D)**], anti–IL-4 [5 μg/ml **(A)** or 2 μg/ml **(D)**], IL-6 (20 ng/ml), and TGFβ (2 ng/ml) for 72 h in the presence of vehicle or daurinol. Three days later, the cells were stained with antibodies to CD4, IL-17, and FoxP3. **(A)** A plot from one representative experiment shows the frequencies of IL-17+, CD25+, and FoxP3+ cells among CD+ T cells. **(B)** Mean ± SD values are presented in the form of a histogram. Data are representative of four independent experiments with similar results. **(C)** IL-17 levels in culture supernatants shown in **(A,B)** were measured by ELISA. **(D)** CD25+, Foxp3+, or IL-17+ cells among CFSE-labeled proliferating CD4+ T cells cultured under the Th17-skewing condition were analyzed by flow cytometry. **(E)** Expression of Treg-related genes (*Foxp3, TGF-*β, *SOCS3*; upper panel) and Th17-related genes (*IL-17, ROR*γ*t, HIF-1*α; lower panel) in the experiments shown in **(D)** were determined by real-time PCR. Data were obtained from three independent experiments, and values are represented as the mean ± SD. *p < 0.05, **p < 0.01, ***p < 0.001, ****p < 0.0001. –, untreated; Veh, vehicle-treated (DMSO); Dau, daurinol.

First, we examined the effects of daurinol on Th17 and Treg differentiation. CD4+ T cells isolated from normal DBA/1J mice were cultured under the Th17-polarizing condition (as described in section Materials and Methods) in the presence or absence of daurinol (concentration ranging from 0.5 to 5 μM) for 72 h. Flow cytometry demonstrated that daurinol treatment of murine CD4+ T cells inhibited Th17 differentiation and promoted Treg differentiation (Figures 1A,B). IL-17 production in culture supernatants of daurinol-treated cells was significantly inhibited in a dose-dependent manner, compared with that in culture supernatants of vehicle-treated cells (Figure 1C).

Next, we analyzed the populations of CD25+, Foxp3+, and IL-17+ cells among CFSE-labeled proliferating T cells cultured under the Th17-skewing condition. As expected, IL-17+ cells among the CD4+ T cells were inhibited by daurinol. Although CD25 is expressed upon activation of CD4+ T cells (20) and is an accepted surface marker of Tregs (21), Foxp3 is the critical master regulator of immunoregulatory function of Tregs and their development (22). Interestingly, daurinol treatment decreased the percentage of CD25+ cells among proliferating CD4+ T cells but increased that of Foxp3+ cells (Figure 1D).

Daurinol treatment increased the mRNA levels of Treg-related molecules (Foxp3, TGF-β, and SOCS3) in murine CD4+ T cells cultured under the Th17-skewing condition (Figure 1E). Hypoxia-inducible factor-1α (HIF-1α), a master transcription factor of hypoxia-inducible genes, plays a crucial role in the balance between Th17 and Treg cells. It directly promotes Th17 differentiation via activation of RORγt, the key transcription factor for Th17 cell differentiation (23), and reciprocally suppresses Treg differentiation by stimulating Foxp3 degradation (24). Daurinol treatment in murine CD4+ T cell decreased the mRNA expression of Th17-related molecules including IL-17, RORγt, and HIF-1α (Figure 1E).

Since splenic CD4+ T cells also contain CD62– memory T cells, the differentiation from splenic total CD4+ T cells and CD62+ naïve T cells into Th17 cells are different. Thus, Th17 differentiation experiments were conducted using only isolated CD44-CD62+ naïve T cells in order to more selectively identify the potential of daurinol during Th17 differentiation. We confirmed that daurinol also induced Th17–Treg reciprocal regulation in a dose-dependent manner (Figure S1A) and attenuated IL-17 production in naïve CD4+ T cells (Figure S1B). To elucidate the Th17–Treg-modulating mechanisms of daurinol, the mRNA expression levels of immunoregulatory mediators expressed in Tregs were analyzed by real-time PCR. Under the Th17-polarizing condition, daurinol treatment (2 μM) of murine splenic CD4+ T cells significantly induced mRNA expression of *Igfbp4, Sell, Nt5e, IL-7R*, and neuropilin-1 (*Nrp1*) (Figure S2). Taken together, these data suggest that the immunoregulatory properties of daurinol occurs through reciprocal regulation of Th17 and Treg differentiation from naïve CD4+ T cells and promotion of gene expression of immunoregulatory mediators of Tregs.

### Nrp1-Dependent Treg Induction by Daurinol

Nrp1 has been identified as a Treg-expressing marker at least in murine T cells (25). Nrp1-induced transcriptome augments Treg stability by promoting survival factor and inhibiting terminal differentiation (26). Nrp1 stabilizes Treg function by potentiating its downstream target, phosphatase and tensin homolog (PTEN) activity (26). PTEN is a negative regulator of the Akt–mTOR signaling axis in T cells (27). PTEN-induced suppression of Akt–mTOR activity helps to maintain Treg function, homeostasis, and stability by augmenting Foxp3 expression (27–29). Based on this rationale, we next examined whether the daurinol treatment could alter the abovementioned signaling pathways that are pivotal for Treg differentiation and stabilization in CD4+ T cells.

**Figure 2:**
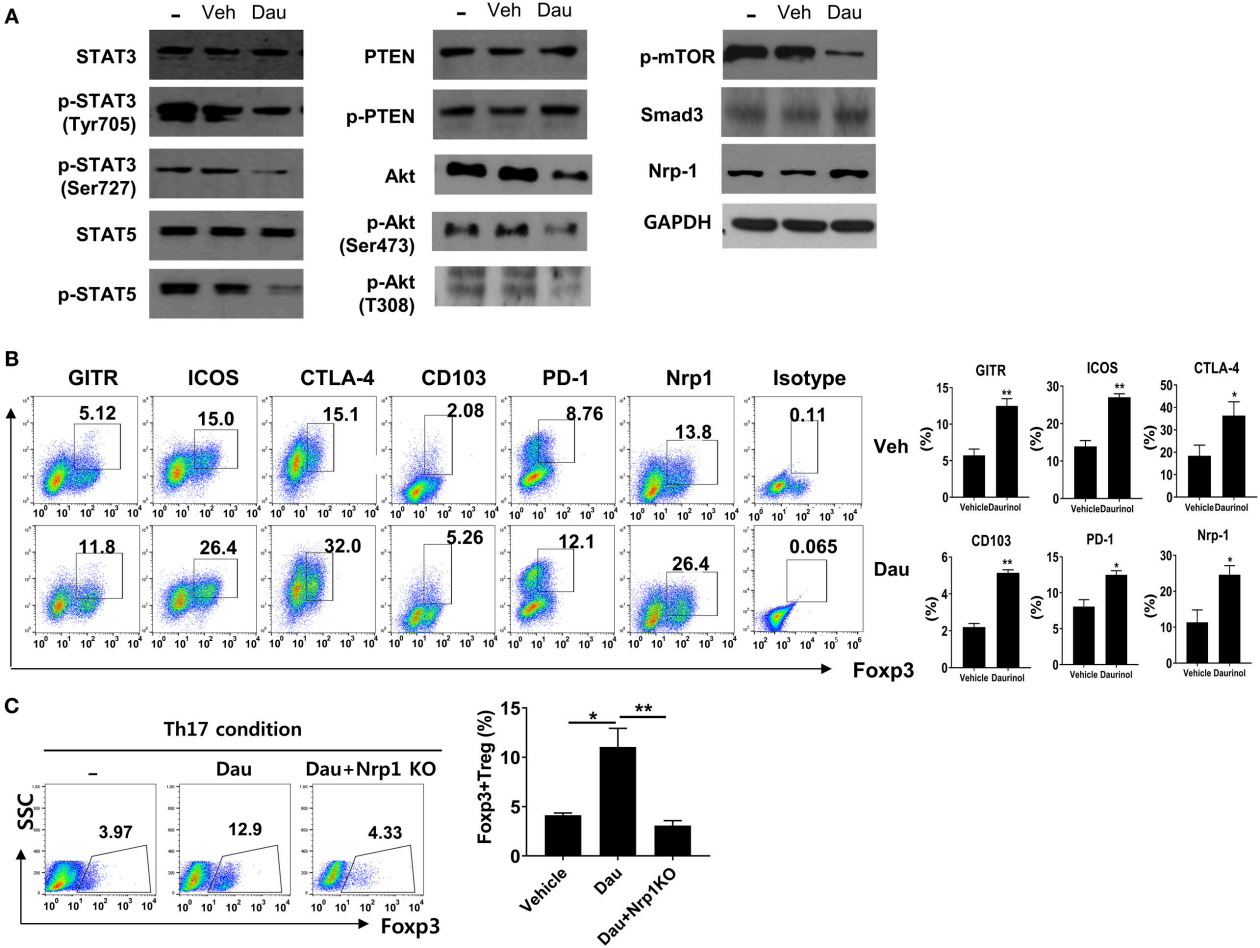
Daurinol induce the Neuropilin-1+Foxp3 Treg cell expansion. **(A)** Mouse splenic CD4+ T cells were cultured under the Th17-skewing condition for 72 h in the presence or absence of daurinol (2 μM). Cell lysates were analyzed by Western blotting to detect total and phosphorylated forms of STAT3 (pSTAT3), STAT5 (pSTAT5), PTEN (pPTEN), Akt (pAkt), and pmTOR, Nrp1, and Smad3. **(B)** The cell populations with the Treg phenotype (GITR+, ICOS+, CTLA-4+, CD103+, PD-1+, and Nrp1+ cells) after daurinol (2 μM) treatment were analyzed by flow cytometry. **(C)** *Nrp1* was deleted in CD4+ T cells using the CRISPR–Cas9 system. Naïve CD4+ T cells and *Nrp1*-deleted cells were cultured under the Th17-polarizing condition in the presence or absence of daurinol. The populations of Foxp3+ T cells were analyzed by flow cytometry. Data were obtained from three independent experiments, and values are represented as the mean ± SD. *p < 0.05, **p < 0.01.

Total CD4+ T cells isolated from mouse spleens were cultured under the Th17-polarizing condition for 3 days. The levels of total and phosphorylated forms of STAT3 (pSTAT3, as a transcriptional factor for Th17 cells), STAT5 (as a transcriptional factor for Tregs), PTEN, and Akt, and phosphorylated mTOR, Smad3 and Nrp1 activity were evaluated by Western blotting in cells treated with or without 2 μM daurinol. Compared with vehicle-treated cells, daurinol treatment markedly attenuated the expression levels of the phosphorylated forms of STAT3 (both Tyr705 and Ser727), Akt (both Ser473 and T308), and mTOR but reciprocally augmented pPTEN, Smad3, and Nrp1 activity (Figure 2A). Interestingly, pSTAT5 activity was inhibited by daurinol treatment (Figure 2A). Although STAT5 plays as a major driver of differentiation and homeostasis of Treg cells, STAT5-independent pathway such as mTOR have a negative impact on Treg cells (30). Previous many studies have demonstrated that specific loss of mTOR activity and mTOR inhibitor (such as rapamycin) treatment resulted in loss of Th1, Th2, and Th17 cells, while enhancing Treg differentiation, implying the selective role of mTOR during Treg differentiation (31–33). Thus, we assumed that daurinol can promote Treg differentiation by Smad3 activity which was promoted by inhibition of mTOR activity.

We examined whether daurinol treatment increased the T cell populations expressing Treg markers such as GITR, ICOS, CTLA-4, CD103, PD-1, and Nrp1. Flow cytometry showed that the percentages of CD4+Foxp3+ T cells expressing Treg markers were increased by daurinol in CD4+ T cells cultured under the Th17-skewing condition; this supports the Treg-inducing property of daurinol (Figure 2B). Next, the CRISPR–Cas9 system was used to determine whether the Treg-inducing property of daurinol is dependent on Nrp1. By applying CRISPR–Cas9 system, Nrp1 activity was effective reduced by about 50% in murine CD4+ T cells (data not shown). The Foxp3+ Treg-inducing property induced by daurinol was diminished by *Nrp1* silencing (Figure 2C). Taken together, these findings suggest that daurinol induces the differentiation of Foxp3+ Tregs and that this effect depends on the Nrp1–PTEN–Akt–mTOR signaling axis.

### Association Between Treg Induction by Daurinol and Decreased Aerobic Glycolysis

**Figure 3:**
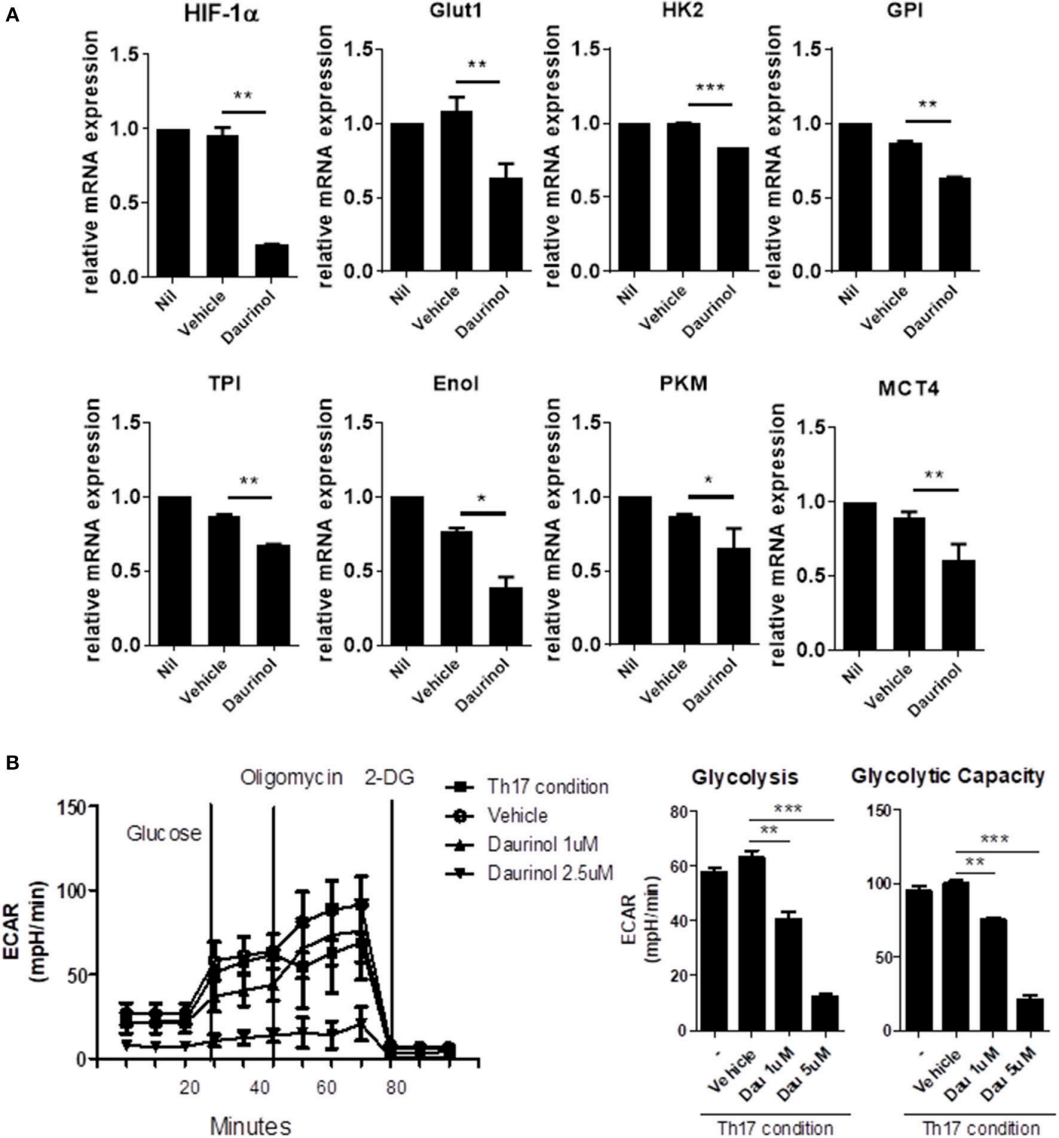
Daurinol inhibits aerobic glycolysis in CD4+T cells. **(A)** The expression levels of aerobic glycolysis-associated genes were determined by real-time PCR in murine CD4+ T cells cultured under the Th17-polarizing condition in the presence of absence of daurinol (2 μM). **(B)** ECAR in CD4+ T cells cultured under the Th17-polarizing condition in the presence or absence of daurinol. The data are representative of three independent experiments and are expressed as the mean ± SD. *p < 0.05, **p < 0.01, ***p < 0.001.

The activation of effector T cells, including Th1 and Th17 cells from naïve T cells, is accompanied by a metabolic switch to aerobic glycolysis to fuel the energy demands of the process (34). By contrast, Tregs have a high fatty-acid oxidation metabolic rate (35). HIF-1α is a key transcription factor that orchestrates the expression of glycolytic enzymes, thereby modulating the differentiation of Th17 and Tregs (36). To identify whether the reciprocal regulation of Th17/Treg cells by daurinol is associated with metabolic switch, we measured glycolytic activity in murine CD4+ T cells cultured under the Th17-polarizing condition. To determine the influence of daurinol on glycolysis, the changes in aerobic glycolysis-associated mediators were analyzed in CD4+ T cells. Real-time PCR analysis of murine CD4+ T cells cultured under the Th17-polarizing condition revealed that daurinol treatment downregulated genes encoding various molecules involved in aerobic glycolysis, such as *Glut1* (glucose transport 1), *MCT4* (monocarboxylic acid transporter member 4), *HK2* (hexokinase 2), *GPI* (glucose-6-phosphate isomerase), *TPI* (triosephosphate isomerase), *Eno1* (enolase 1), and *PKM* (pyruvate kinase muscle), compared with those of vehicle-treated cells (Figure 3A). Glut1 is a transporter for glucose uptake and is rapidly induced following T cell activation and plays a pivotal role in effector T cells (37). MCT4 is a plasma membrane transporter for the lactate exporter and is involved in aerobic glycolysis (38).

Next, we measured the extracellular acidification rate (ECAR) to investigate whether daurinol could change the degree of glycolysis in CD4+ T cells. Daurinol decreased glycolysis in CD4+ T cells cultured in the Th17-skewing condition (Figure 3B), which suggests that Treg induction by daurinol treatment may occur through the metabolic changes.

### Increased Differentiation and Stabilization of Tregs by Daurinol Through Foxp3 Hypomethylation

**Figure 4:**
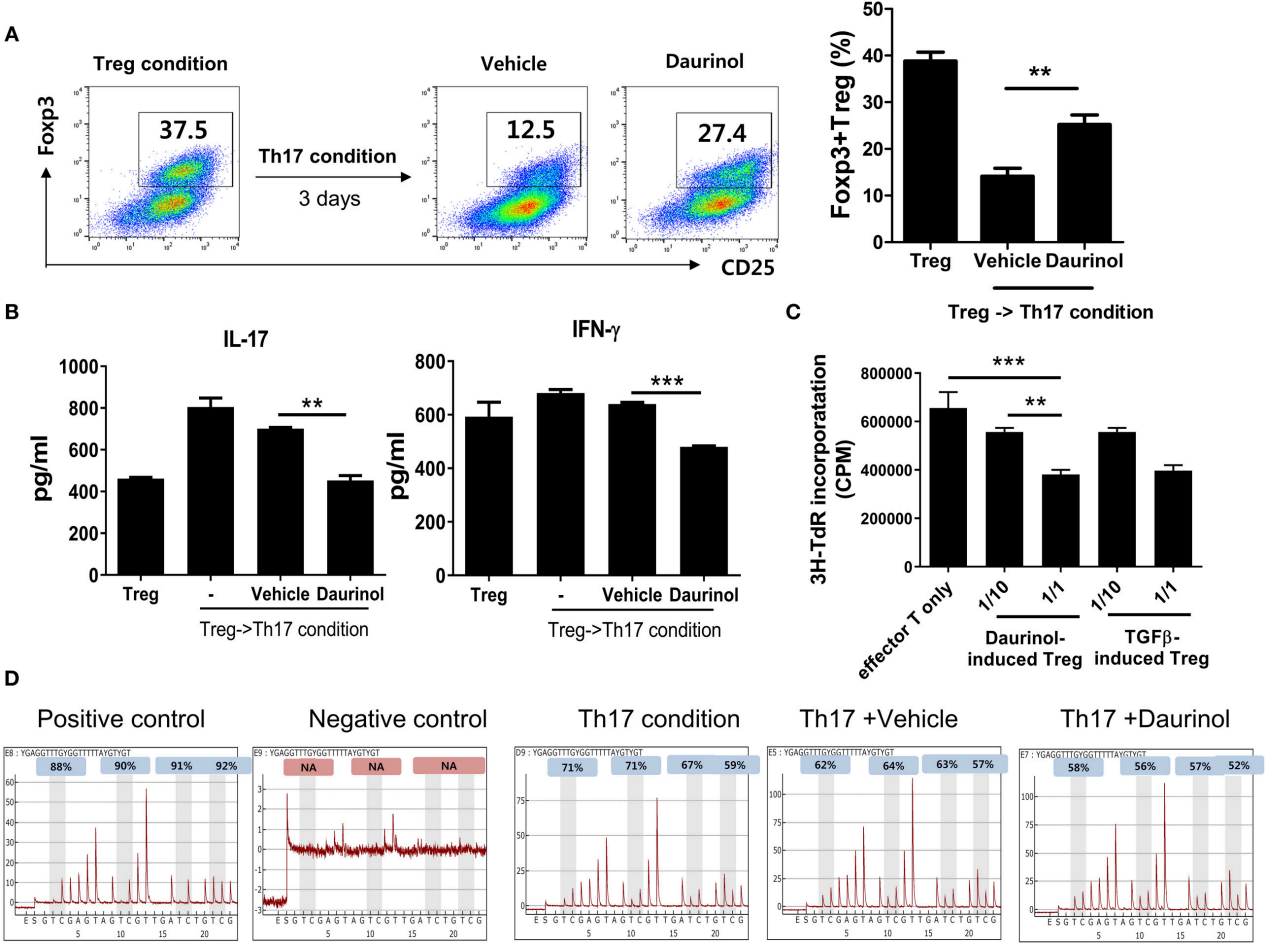
Stable immunoregulatory potential of daurinol-induced Tregs associated with Foxp3 hypomethylation. **(A)** Murine CD4+ T cells were cultured under the Treg-inducing condition and cultured for a further 72 h under the Th17-inducing condition in the presence or absence of daurinol (2 μM). The CD4+CD25+Foxp3+ Treg population was analyzed by flow cytometry. **(B)** IL-17 and IFN-γ levels in culture supernatants from cells described in **(A)** were measured by ELISA. –, untreated **(C)** T cell stimulatory capacity of daurinol-induced Tregs. Anti-CD3-stimulated CD4+ T cells were cultured with daurinol-induced Tregs at different Treg:effector T cell ratios for 3 days. T cell proliferation was determined on day 3 of culture by measuring the incorporation of ^3^H-thymidine. Data are presented as mean ± SD (bars) of triplicates. **p < 0.01, ***p < 0.001. **(D)** Representative pyrograms showing hypomethylation of Foxp3 in CD4+ T cells cultured under the Th17-skewing condition with daurinol treatment.

Next, to confirm the function of daurinol in terms of Treg stabilization, murine CD4+ T cells were cultured under the Treg-polarizing condition for 72 h and then further cultured under the Th17-polarizing condition for 72 h. The population of Tregs induced under the Treg-polarizing condition decreased after culture under the Th17-skewing condition. Interestingly, daurinol treatment in murine CD4+ T cells prevented this decrease in Treg populations (Figure 4A). The concentrations of IL-17 and IFN-γ in culture supernatants were measured by ELISA. The production of IL-17 and IFN-γ was inhibited in daurinol-treated T cells compared with vehicle-treated T cells (Figure 4B).

Next, we studied whether daurinol-induce Tregs have an immunoregulatory function by inhibiting the proliferation of anti-CD3-stimulated CD4+ T cells. The proliferative response of anti-CD3-stimulated CD4+ T cells cultured with daurinol-induced Tregs (effector T cells alone, 1:10, 1:1) was evaluated by measuring [^3^H] thymidine incorporation. Daurinol-induced Tregs suppressed the proliferation of effector T cells in a ratio-dependent manner, and these cells showed the same suppression effect as TGFβ-induced Tregs (Figure 4C), which suggests that the daurinol stimulated the differentiation of Tregs and their immunoregulatory function. Foxp3 is a major transcription factor for Treg induction and its maintenance is essential for the control of inflammation. The hypomethylated region within *Foxp3* is considered to be the hallmark of stable Tregs (39, 40). Therefore, we examined whether daurinol can directly modulate *Foxp3* and, if so, the regulatory mechanism. To analyze the methylation density of the promoter region Foxp3 gene by daurinol, we conducted pyrosequencing of bisulfite-modified genomic DNA from CpG island at promoter regions of Foxp3. Daurinol treatment of CD4+ T cells cultured under the Th17-polarizing condition induced decreased methylation of CpG sites at Foxp3 promoter regions, which stimulated the differentiation and increased the stability of Tregs (Figure 4D).

### Attenuation of the Development of Inflammatory Arthritis by Daurinol in a Dose-Dependent Manner

**Figure 5:**
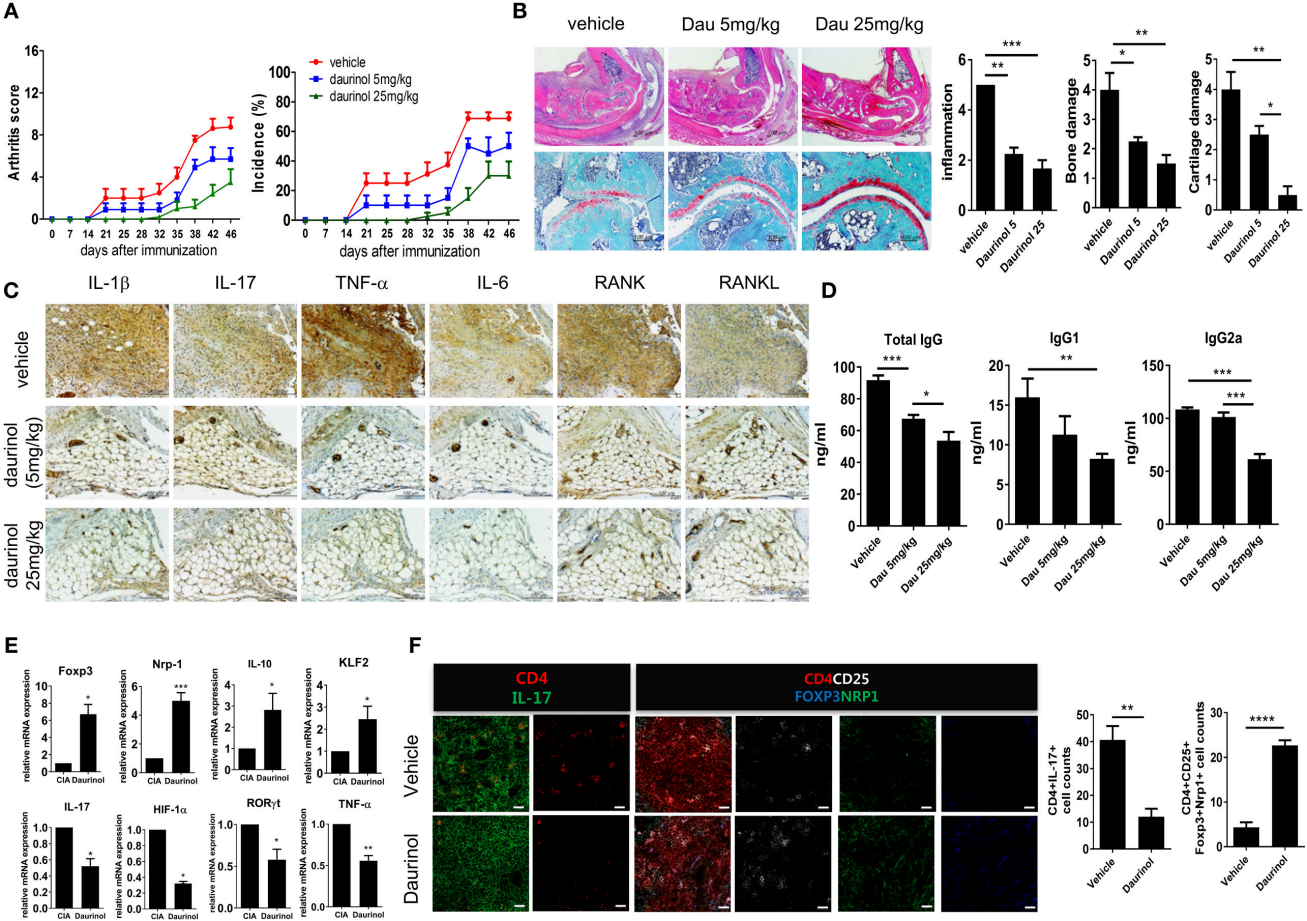
*In vivo* therapeutic effects of oral administration of daurinol on the development of autoimmune arthritis. Three weeks after immunization with type II collagen (CII), mice with CII-induced arthritis (CIA) were orally administered vehicle or daurinol (5 mg/kg or 25 mg/kg) once every 2 days for 3 weeks. **(A)** Clinical scores for arthritis (left) and incidence of arthritis (right) are shown for each treatment group over time (representative results from one of two independent experiments). **(B)** At 46 days after the first CII immunization, tissue sections were obtained from the ankle joints of mice with CIA and stained with hematoxylin and eosin (H&E; original magnification ×40) and Safranin O (original magnification ×200) to examine the severity of arthritis (left). Histological scores for inflammation, bone damage, and cartilage damage were determined (right). **(C)** Synovial tissue sections of ankle joints from each group of mice were stained with antibodies to IL-1β, IL-17, TNF-α, IL-6, RANK, and RANKL. **(D)** Concentrations of total and CII-specific IgG1 and IgG2a in the serum of mice from each group were measured by ELISA. Data show the mean ± SD (bars) for six mice per group. **(E)** RNA was extracted from splenic CD4+ T cells from vehicle- or daurinol (25 mg/kg)-treated CIA mice and analyzed by real-time PCR for the expression of *Foxp3, Nrp1, IL-19, KLF2, IL-17, HIF-1*α, *ROR*γ*t*, and *TNF-*α mRNA. Bars show the mean ± SD mRNA expression relative to that of GAPDH for six mice per group from two independent experiments. **(F)** Left, Spleens were examined by immunofluorescence staining with monoclonal antibodies against CD4 (red), IL-17 (green), CD25 (blue), Foxp3 (yellow), and Nrp1 (green). Original magnification ×400. Scale bar represents 20 μM. *Right*, CD4+IL-17+ Th17 cells and CD4+CD25+Fop3+Nrp1+ Tregs were enumerated visually at higher magnification as projected on a screen, with each confocal image representative of four fields of view. Values represent the mean ± SD for six mice per group from at least three independent experiments. *p < 0.05, **p < 0.01, ***p < 0.001, ****p < 0.0001.

We investigated whether daurinol could suppress inflammation and joint destruction in an experimental murine model of RA (CIA). Daurinol (5 mg/kg or 25 mg/kg) was administered orally once every 2 days for 3 weeks from day 21 after primary immunization with CII emulsified in Freund's complete adjuvant. Daurinol ameliorated arthritis severity and incidence compared with vehicle-treated CIA mice in a dose-dependent manner (Figure 5A). Histological sections of hind paw joints showed that daurinol treatment in CIA mice attenuated the severity of inflammation, cartilage damage, and bone erosion, as investigated by hematoxylin–eosin (H&E) and Safranin O staining (Figure 5B). IL-1β, IL-17, TNF-α, and IL-6 are considered to be proinflammatory cytokines that are implicated in the pathogenesis of RA (41). The role of the receptor activator of nuclear factor κB ligand (RANKL)/RANK system has been extensively studied in joint destruction in RA and is a major treatment target in RA. Compared with joints of vehicle-treated mice with CIA, joints of daurinol-treated mice with CIA had significantly fewer cell populations expressing IL-1β, IL-17, TNF-α, IL-6, RANKL, and RANK (Figure 5C). Serum levels of CII-specific IgG, IgG1, and IgG2a antibodies were lower in daurinol-treated mice than in vehicle-treated mice, and the effects were dose dependent (Figure 5D).

IL-10, one of the anti-inflammatory cytokines, restrains the Th17-medicated inflammatory process (42). IL-10 signaling is also pivotal for maintaining the immunoregulatory function of Tregs (43). Krüppel-like factor 2 (KLF2) promotes Treg generation and function through Foxp3 induction (44). We analyzed the mRNA expression of factors associated with the differentiation and function of Th17 and Tregs in splenic CD4+ T cells isolated from each group of mice. mRNA expression levels of Treg factors in the splenic T cells were significantly higher after daurinol treatment (25 mg/kg) compared with cells from CIA mice treated with vehicle. By contrast, the mRNA expression of Th17 factors was decreased by daurinol treatment (Figure 5E). Confocal analysis showed significantly suppressed IL-17+ and reciprocally augmented CD25+Foxp3+Nrp1+ cell populations among splenic CD4+ cells after daurinol treatment in CIA mice (Figure 5F).

Next, we confirmed the anti-inflammatory effect of intraperitoneal (IP) administration of daurinol. Daurinol (20 mg/kg) was administered intraperitoneally once every 2 days for 3 weeks from day 21 after CII immunization. IP daurinol treatment significantly reduced the arthritis severity and incidence compared with those observed in vehicle-treated CIA mice (Figure S3A). Serum levels of CII-specific IgG, IgG1, and IgG2a antibodies were lower in daurinol-treated mice than in vehicle-treated mice (Figure S3B). Histological sections of ankle joints stained with H&E, Safranin O, and toluidine blue showed less severe arthritis in IP daurinol-treated CIA mice compared with vehicle-treated mice (Figure S3C). Compared with joints of vehicle-treated mice, joints of IP daurinol-treated mice with CIA exhibited smaller populations of cells expressing TNF-α, IL-17, IL-6, and IL-1β (Figure S3D).

To determine whether the populations of Th17, Th1, Th2, and Tregs were altered in daurinol-treated mice with CIA, we used flow cytometry to analyze IL-17-, IFN-γ-, IL-4-, and Foxp3-expressing cells among CD4+ cells in the spleens from mice with CIA. Spleens from daurinol-treated CIA mice showed fewer Th17, Th1, and Th2 cells and a reciprocal increase in the number of Foxp3-expressing Tregs compared with spleens from vehicle-treated mice (Figure S4A). IP daurinol treatment increased the populations of CD4+ cells expressing Treg markers, such as GITR, ICOS, CD103, CTLA-4, or PD-1 in CIA mice (Figure S4B). Confocal immunostaining of spleen tissue sections also showed significantly decreased populations of CD4+ T cells that was expressing pSTAT3Ser727, and a significantly increased population of CD4+CD25+Foxp3+ T cells (Figure S5).

### Inhibition of Osteoclastogenesis by Daurinol *in vitro* and *in vivo*

Pathologically enhanced osteoclast activation is an important therapeutic target, which contribute to progressive joint damage in RA patients. Since both oral and intraperitoneal administration of daurinol showed a significant anti-arthritic effects *in vivo*, we tried to confirm whether daurinol affects not only T cell subset differentiation but also osteoclastogenesis. For this purpose, BMM cells isolated from normal DBA/1J mice were cultured with M-CSF and RANKL in the presence or absence of daurinol (2 μM). TRAP staining showed that daurinol treatment significantly inhibited *in vitro* osteoclastogenesis in BMMs compared with vehicle-treated cells (Figure S6A). We next tried to identify the pro-osteoclastogenic factors affected by *in vivo* daurinol treatment. BMM cells were isolated from daurinol-treated CIA mice or vehicle-treated CIA mice and were cultured in the presence of M-CSF and RANKL. Real-time PCR was used to analyze the levels of mRNA for osteoclast markers, including *TRAP, MMP9, carbonic anhydrase II, calcitonin receptor, and Itgb3* (integrin β3) in the cells. mRNA transcript levels of osteoclastogenesis markers were also significantly lower in daurinol-treated CIA mice than in the vehicle-treated animals (Figure S6B). This finding suggests that daurinol ameliorates CIA by reducing osteoclastogenesis in mice.

### Daurinol Upregulates Treg Cell Through Nrp1-PTEN Signaling and Reciprocal Inhibition of Th17 Cell in Human CD4+ T Cells

**Figure 6:**
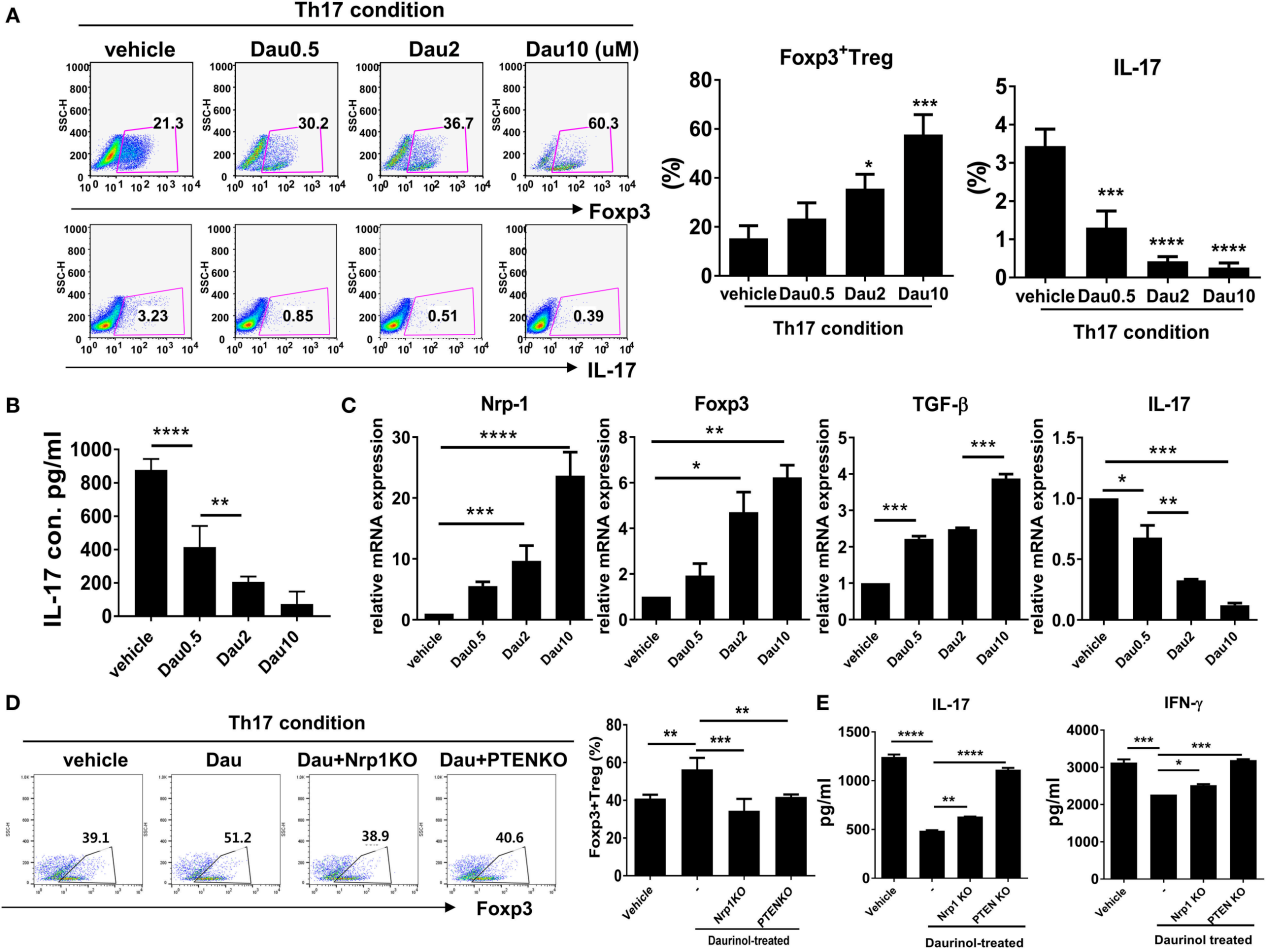
Reciprocal regulation of Nrp1 and PTEN signaling-dependent human Th17 and Treg differentiation by daurinol. Human CD4+ T cells from peripheral blood mononuclear cells were treated with daurinol (dose, 0.5–10 μM) or vehicle under the Th17 cell-polarizing condition for 3 days. **(A)** The populations of CD4+ Foxp3+ cells and CD4+Foxp3+ cells were analyzed by flow cytometry. Th17 cell differentiation was inhibited by daurinol treatment in a dose-dependent manner, whereas Treg cell differentiation was reciprocally increased. **(B)** IL-17 levels in culture supernatants were measured by ELISA. **(C)** Expression of *Nrp1, Foxp3, TGF-*β, and *IL-17* mRNA was determined by real-time PCR. **(D)** The populations of Foxp3+CD4+ cells altered by *Nrp1* and *PTEN* knockdown in the CRISPR–Cas9 system in human CD4+ T cells were analyzed by flow cytometry. Foxp3+CD4+ T cell differentiation was inhibited by attenuating the expression of *Nrp1* and *PTEN* compared with untreated cells. **(E)** IL-17 and IFN-γ levels in culture supernatants in the experiments shown in **(D)** were measured by ELISA. Data were obtained from at least three independent experiments, and values are represented as the mean ± SD *p < 0.05, **p < 0.01, ***p < 0.001, ****p < 0.0001.

We next investigated the effects of daurinol on human CD4+ T cells isolated from PBMCs obtained from normal healthy volunteers. Purified CD4+ T cells were cultured under the Th17-polarizing condition in the presence or absence of daurinol (at doses of 0.5–10 μM). Daurinol treatment significantly increased the Foxp3+ Treg cell population in a dose-dependent manner but suppressed Th17 cell differentiation (Figure 6A). Daurinol also reduced the IL-17 level in the culture supernatant (Figure 6B). We measured the mRNA levels of Treg- and Th17-related molecules in the cells. Daurinol-induced Treg induction and Th17 suppression were associated with increased levels of mRNA encoding *Nrp1, Foxp3*, and *Tgfb*, and with decreased *Il17* mRNA level (Figure 6C). We next used the CRISPR–Cas9 system to confirm that Treg induction effect by daurinol is dependent on Nrp1 and PTEN. By applying CRISPR–Cas9 system, Nrp1, and PTEN protein activity was effective reduced by about 50% in human CD4+ T cells (Figure S7). The results showed that Treg induction by daurinol in human CD4+ T cells was also dependent on Nrp1–PTEN signal (Figure 6D), which suggests that human T cell reactions to daurinol correspond to those observed in murine CD4+ T cells. The decreased production of IL-17 and IFN-γ induced by daurinol treatment was attenuated by knockdown of *Nrp1* and *Pten* (Figure 6E).

## Discussion

Treg-induced immune tolerance has emerged as an attractive strategy in RA treatment. However, the ability to generate a sufficient number of Tregs with functional stability by *in vitro* expansion remains an issue that hinders the clinical exploitation of these cells in autoimmune diseases including RA. In this study, we first confirmed that daurinol diverted the differentiation of human and murine CD4+ T cells toward a Treg phenotype, even under Th17-polarizing conditions, and increased Treg stability through Nrp1–PTEN–Foxp3 signaling. Interestingly, the optimal and stable immunoregulatory function of Tregs induced by daurinol is associated with hypomethylation of the lineage-specific transcription factor Foxp3. The Tregs induced by daurinol treatment showed significantly reduced aerobic glycolysis. The change in the metabolic profile may explain the reduced populations of effector T cells including Th1, Th2, and Th17 cells, and reciprocally augmented Treg subsets in daurinol-treated CIA mice compared with vehicle-treated animals. Here, we found that systemic administration of daurinol (oral and IP administration) effectively reduced the clinical and histological scores in a murine model of RA. The Treg-inducing property by *in vivo* daurinol treatment in CIA mice was associated with significant induction of Nrp1 and Foxp3 expression. In addition, daurinol significantly inhibited osteoclast differentiation and related gene expression, which suggests that daurinol has an inhibitory effect on bone destruction. However, one of the limitation of our study is that the presentation of inherent mechanisms to explain the anti-inflammatory and immunoregulatory effects of daurinol shown *in vivo* is fairly limited.

One reasonable treatment strategy for autoimmune diseases under a new paradigm may involve optimizing the immunoregulatory function of Tregs. Some trials have targeted Tregs in autoimmune diseases including graft-vs.-host disease (45), SLE (46), and RA (47). However, few studies have targeted the epigenetic stabilization of Foxp3, a key transcription factor of Tregs. The ways by which Tregs maintain their lineage stability and immunosuppressive function include multiple epigenetic changes (48). Epigenetic changes including DNA methylation affect cell differentiation and lineage stabilization at the level of transcription (49, 50). In the research field of Treg function, several studies have been published to support the claim that demethylation of Foxp3 gene promoter sites mainly contributes to their immunoregulatory function and development of a stable suppressor cell lineage (40, 51). Through our present study, we first proved that daurinol has immunoregulatory potential via the epigenetic change (DNA demethylation) of Foxp3 promoter region, rather than the expression control of transcription factor STAT5.

We assumed that daurinol has shown Treg-induction effects through modulation of mTOR/Akt signaling activity. The sustained expression of Foxp3 is important for maintenance of Treg lineage. Foxp3 retains the immunoregulatory function of Tregs and blocks the transition of Tregs into effector T cells, such as Th1 and Th17 cells (52). Previous study demonstrated clearly that Foxp3-negative Tregs lose their suppressive function and assume an inflammatory phenotype (53). The forkhead box protein O (FoxO) is a transcription factor that induces Foxp3 gene expression by enhancing Foxp3 promoter region, thereby contributes to Tregs stabilization and maintaining their immunoregulatory function (54). Interestingly, Akt-induced phosphorylation of FoxO protein triggers the translocation of FoxO protein form nucleus to cytoplasm, which ultimately results in the suppression of Foxp3 expression (55). Although this study could not clarify the underlying mechanisms by which mTOR/Akt axis regulation by daurinol affected the subcellular location of FoxO protein, we presumed that mTOR/Akt/FoxO axis regulation by daurinol induced Foxp3 promoter activity, which ultimately resulted in Tregs induction. Promoted Treg function driven by sustained Foxp3 expression by daurinol may be a novel treatment strategy for autoimmune diseases that involve impaired Treg function, such as RA and SLE.

Our study provides the first evidence that daurinol can regulate the differentiation of Tregs and stabilize their immunoregulatory function. Our findings suggest that daurinol can regulate Th17 differentiation by STAT3 inhibition while stimulating Treg differentiation and stabilizing Tregs through Nrp1–PTEN–Foxp3 signaling. By hypomethylation of Foxp3, a Treg lineage-specific transcription factor, daurinol concurrently stimulates Treg differentiation while suppressing the Th17 population.

## Ethics Statement

The protocols used in this study were approved by the Animal Care and Use Committee of the Catholic University of Korea.

## Author Contributions

All authors were involved in drafting the article or revising it critically for important intellectual content, and all authors approved the final version to be published. M-LC and DS had full access to all of the data in the study and takes responsibility for the integrity of the data and the accuracy of the data analysis. M-JP, S-JM, DS, and M-LC contributed to study conception, study design, data acquisition, analysis, and interpretation and drafted the manuscript. E-JL, E-KK, J-AB, S-YK, SHL, D-SK, KJ, and JC contributed to data acquisition, analysis, and interpretation. SHL, J-KM, and S-HP contributed to analysis and interpretation of data.

### Conflict of Interest Statement

The authors declare that the research was conducted in the absence of any commercial or financial relationships that could be construed as a potential conflict of interest.
