# Supplementary material for: Daurinol Attenuates Autoimmune Arthritis via Stabilization of Nrp1–PTEN–Foxp3 Signaling in Regulatory T Cells
Source: Front Immunol. 2019 Jul 17;10:1526. doi: 10.3389/fimmu.2019.01526 (PMC6651269; doi:10.3389/fimmu.2019.01526)
Supplement: Supplementary file 1 [file Data_Sheet_1.docx]

**Daurinol attenuates autoimmune arthritis via stabilization of Nrp1–PTEN–Foxp3 signaling in Regulatory T cells**

**Park MJ et al.**

**SI Materials and Methods**

**Mice**

DBA/1J and C57BL/6 (B6) mice, 8–10 weeks of age, were purchased from OrientBio (Sungnam, Korea) and were maintained under specific-pathogen-free conditions in an animal facility. The protocols used in this study were approved by the Animal Care and Use Committee of the Catholic University of Korea

**Induction of arthritis and daurinol treatment**

Collagen-induced arthritis (CIA) was induced in DBA1/J mice (each group: n = 10). Mice were immunized with 100 μ g of chicken CII (Chondrex Inc., Redmond, WA, USA) dissolved overnight in 0.1N acetic acid (4 mg/ml) in complete Freund’s adjuvant or incomplete Freund’s adjuvant (Chondrex Inc). The immunization was performed intradermally into the base of the tail. The mice were randomly assigned to three experimental groups (n = 10) and treated with daurinol (20 mg/kg) or vehicle by intraperitoneal administration three times a weeks for 3 weeks since 3 weeks after 1^st^ CII immunization.

**Clinical scoring and histological assessment of arthritis**

The onset and severity of arthritis were measured visually twice per week based on the appearance of arthritis, based on the previously described scoring system (1). Arthritic score measurements were performed as follows: 0 = no joint swelling; 1 = slight edema and erythema limited to the foot or ankle; 2 = slight edema and erythema from the ankle to the tarsal bone; 3 = moderate edema and erythema from the ankle to the tarsal bone; and 4 = edema and erythema extending from the ankle to the entire leg, with severe swelling of the wrist or ankle. The final arthritis score was calculated as the sum of scores from all four legs, which were assessed by three independent observers with no knowledge of the experimental groups. The mice were observed twice a week for the onset and severity of joint inflammation for up to end point (day 46) after the initial immunization. Before being sacrificed at 46 days after CIA induction by cervical dislocation, mice were anesthetized using 2–3% isoflurane. At end point, the mice were scarified and hind joint tissues, and spleen were harvested for the further studies. The joints of each mouse were fixed in 10% formalin, decalcified in 10% EDTA, and embedded in paraffin wax for histological analysis. The hind joint tissues from CIA-vehicle and CIA-daurinol groups were subject to histopathological examination such as scoring of inflammation, destruction of cartilage, and bone damage according to published (2, 3) by Hematoxylin-Eosin (H&E) staining, safranin O and toluidine blue staining.

**Measurement of cytokine and IgG levels**

The concentrations of IL-17 in culture supernatants and serum were measured using a sandwich enzyme-linked immunosorbent assay (ELISA Duoset; R&D Systems, Lille, France). Serum levels of IgG, IgG1, and IgG2a antibodies were measured using a commercially available ELISA kit (Bethyl Laboratories).

**Murine and human T cell isolation and differentiation**

To purify mouse splenic or human CD4^+^ T cells, the splenocytes were incubated with CD4-coated magnetic beads and isolated using magnetic-activated cell sorting separation columns (Miltenyi Biotec, Bergisch Gladbach, Germany). Mouse Th17 cell differentiation was induced by treatment with anti-CD3 (0.5 μg/ml); and soluble anti-CD28 (0.5 μg/ml), IL-6 (20 ng/ml) and TGF-β (2 ng/ml), anti-IFN-γ and anti-IL-4 antibodies (each at a concentration of 2 or 5 μg/ml). HumanTh17 cells were stimulated with plate-bound anti-CD3 (0.5 μg/ml); and soluble anti-CD28 (0.5 μg/ml), anti-IFN-γ (2 μg/ml), anti-IL-4 (2 μg/ml), anti-IL-1β (20 ng/ml), and anti-IL-6 (20 ng/ml) for 72 h.

**Real-time polymerase chain reaction (PCR)**

Messenger RNA (mRNA) was extracted using the TRI Reagent (Molecular Research Center, Inc. Cincinnati, OH, USA) according to the manufacturer’s instructions. Complementary DNA was synthesized using a SuperScript Reverse Transcription system (Takara Bio Inc., Otsu, Japan). A LightCycler 2.0 instrument (software version 4.0; Roche Diagnostics, Mannheim, Germany) was used for PCR amplification. All reactions were performed using the LightCycler FastStart DNA Master SYBR Green I mix (Takara Bio Inc.), following the manufacturer’s instructions. Primer sequences are described in Supplementary Table 1 and 2. All mRNA levels were normalized to that of β-actin.

**Flow cytometry**

Mononuclear cells were immunostained with various combinations of the following fluorescence-conjugated antibodies: CD25, CD4, FoxP3, IL-17, CTLA-4, and glucocorticoid-induced tumor necrosis factor receptor (GITR), ICOS, C103, PD-1. These cells were also intracellularly stained with the following antibodies: CTLA-4 (BD Biosciences), IL-17, and FoxP3 (eBioscience). Prior to intracellular staining, cells were restimulated for 4 h with phorbol myristate acetate (25 ng/ml) and ionomycin (250 ng/ml) in the presence of GolgiSTOP (BD Biosciences). Intracellular staining was conducted using a kit (eBioscience), following the manufacturer’s protocol. Flow cytometry was performed using a FACSCalibur instrument (BD Biosciences).

**Confocal microscopy and immunostaining**

Spleen tissues were obtained 46 days after CII immunization, snap-frozen in liquid nitrogen, and stored at –80 °C. Tissue cryosections (7 μm thick) were fixed in 4% (v/v) paraformaldehyde and stained using fluorescein isothiocyanate (FITC)-, phycoerythrin (PE)-, PerCP-Cy5.5-, or allophycocyanin -conjugated monoclonal antibodies to mouse CD4, CD25, pSTAT3 (Ser^727^), IL-17, and Foxp3 (eBioscience). After incubation overnight at 4 °C, stained sections were visualized by confocal microscopy (LSM 510 Meta; Zeiss, Göttingen, Germany).

**Immunohistochemistry**

Immunohistochemistry was performed using the VECTASTAIN ABC kit (Vector Laboratories, Burlingame, CA, USA). Tissues were first incubated with the primary anti-IL-17, anti-TNF-a, anti-IL-1β, and anti-IL-6 antibodies overnight at 48 °C. The primary antibody was detected with a biotinylated secondary linking antibody, followed by incubation with a streptavidin–peroxidase complex for 1 h. The final color product was developed using DAB chromogen (DAKO, Carpinteria, CA, USA).

**Ex vivo and in vitro osteoclastogenesis**

Bone marrow cells from normal mice were cultured overnight, and nonadherent bone marrow cells were harvested and cultured with daurinol in the presence of 10 ng/ml of macrophage colony-stimulating factor (M-CSF) and 20 ng/ml of RANKL. TRAP staining was then performed. TRAP-positive cells with 3 nuclei were defined. as osteoclasts, and the number of osteoclasts was counted. To investigate osteoclast differentiation in vivo, we isolated bone marrow cells from mice with CIA treated with either vehicle and daurinol cultured them with 10ng/ml of M-CSF and 20 ng/ml of RANKL for 3 days. The harvested cell was analyzed with real time PCR.

**Western blotting**

Proteins were separated by sodium dodecyl sulfate polyacrylamide gel electrophoresis (SDS–PAGE) and transferred to nitrocellulose membranes (Amersham Pharmacia Biotech, Buckinghamshire, UK). Membranes were stained with primary antibodies against phosphorylated (active) form of Nrp1, PTEN, Nrp1 (all from Cell Signaling, Danvers, MA, USA), and β-actin. A horseradish peroxidase (HRP)-conjugated secondary antibody was then added.


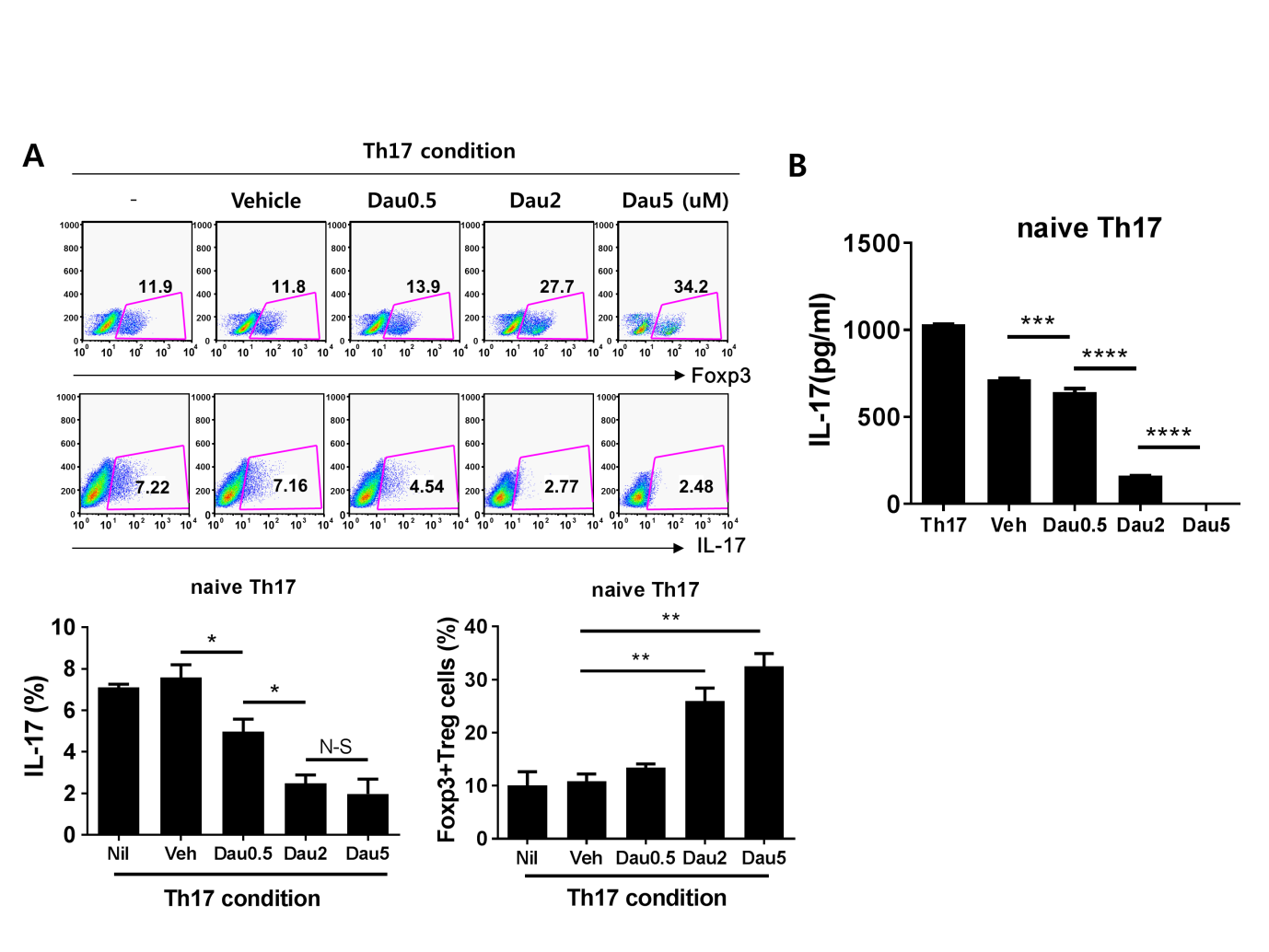


**Supplementary Figure 1. Reciprocal effects of daurinol on Th17 and Treg differentiation in CD44–CD62+ (naïve) CD4+ T cells.** Splenic naïve (CD44–CD62+) CD4+ T cells from DBA/1J mice were cultured under the Th17-inducing condition (cultured with plate-bound anti-CD3 (0.5 μg/ml), soluble anti-CD28 (1 μg/ml), anti-interferon-γ (anti-IFN-γ; 5 μg/ml), anti-IL-4 (5 μg/ml), IL-6 (20 ng/ml), and TGFβ (2 ng/ml) in the presence of vehicle or daurinol. Three days later, the cells were stained with antibodies to IL-17, and Foxp3 among CD25+ cells. **(*A*)** A plot from one representative experiment shows the frequencies of IL-17+ and CD25+Foxp3+ cells among the naïve CD4+ T cells (upper panel). The mean proportion of IL-17+ and Foxp3+ cells among naïve T cells recorded in three independent experiments (lower panel). (***B*)** IL-17 levels in culture supernatants shown in **(A)** were measured by ELISA. Data were obtained from three independent experiments, and values are represented as the mean ± SD (bars). **p* < 0.05, ** *p* < 0.01, *** *p* < 0.001, **** *p* < 0.0001. . ─, untreated; Veh, vehicle-treated (DMSO); Dau, daurinol.


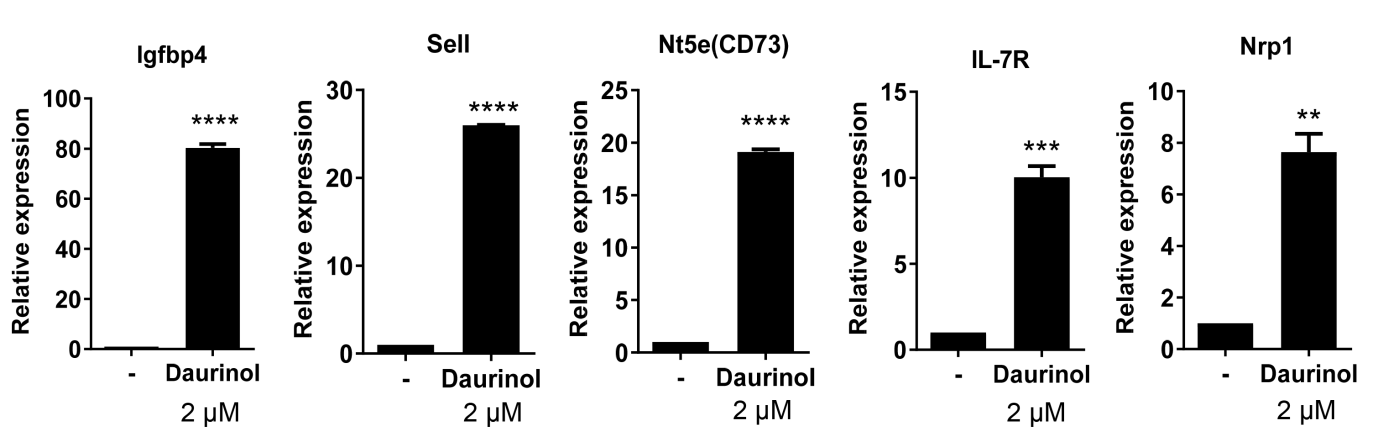


**Supplementary Figure 2. Augmented gene expression of Treg-associated mediators by daurinol treatment.** Murine CD44–CD62+ naïve CD4+ T cells were cultured under the Th17-polarizing condition for 72 h, and the mRNA expression levels of Treg-associated mediators were measured by real-time PCR in the cells. Data were obtained from three independent experiments, and values are represented as the mean ± SD (bars). ** *p* < 0.01, *** *p* < 0.001, **** *p* < 0.0001. ─, untreated


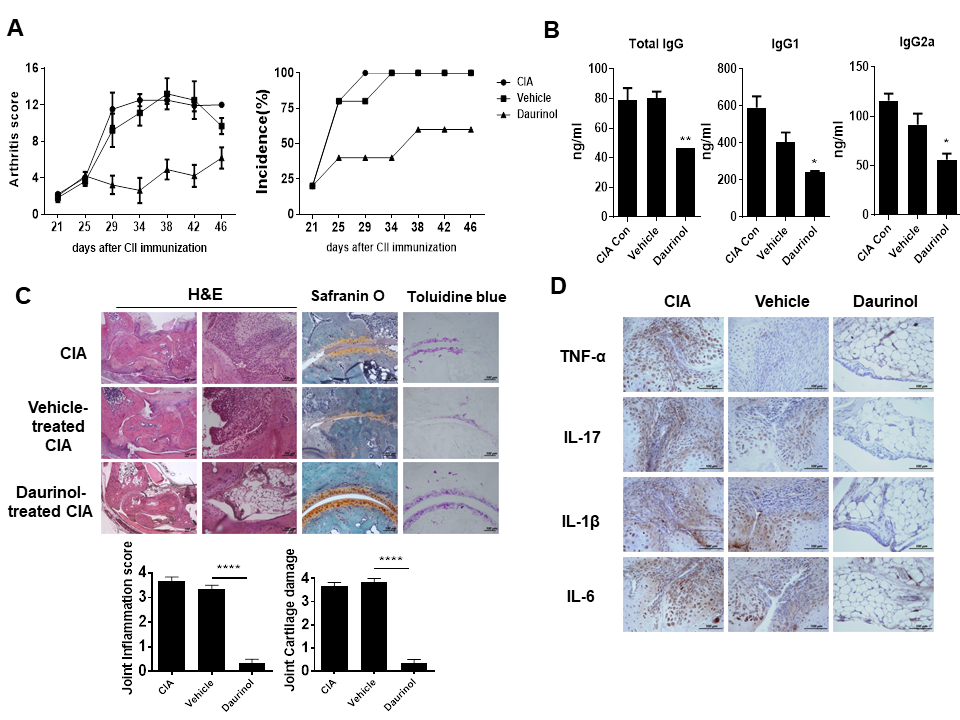


**Supplementary Figure 3. *In vivo* therapeutic effects of intraperitoneal administration of daurinol on the development of autoimmune arthritis.** Three weeks after immunization with type II collagen (CII), mice with CII-induced arthritis (CIA) were intraperitoneally administered vehicle or 20 mg/kg of daurinol once every 2 days for 3 weeks. **(A)** Clinical scores for arthritis (left) and incidence of arthritis (right) are shown for each treatment group over time (representative results from one of two independent experiments). **(B)** Concentrations of total IgG, IgG1 and IgG2a in the serum of mice from each group were measured by ELISA. Data show the mean ± SD (bars) for six mice per group. **(C)** At 46 days after the first CII immunization, tissue sections were obtained from the ankle joints of mice with CIA and stained with hematoxylin and eosin (H&E; original magnification ×40), Safranin O (original magnification ×200), and toluidine blue (original magnification ×200) to examine the severity of arthritis (upper). Histological scores for inflammation and cartilage damage were determined (lower). **(D)** Synovial tissue sections of ankle joints from each group of mice were stained with antibodies to TNF-α, IL-17, IL-1β, and IL-6. Scale bar; 100 μM. * *p* < 0.05, ** *p* < 0.01, **** *p* < 0.0001.


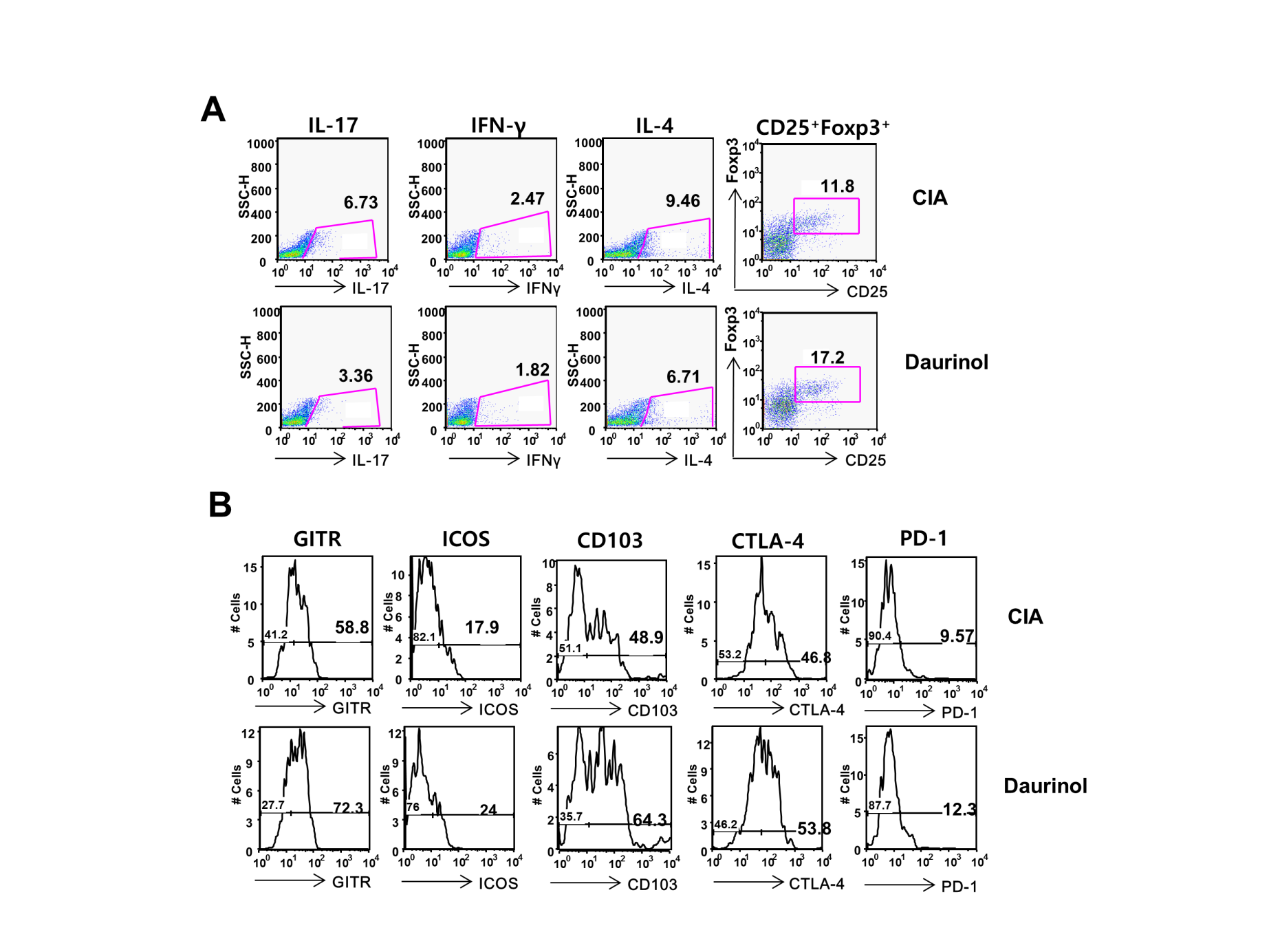


**Supplementary Figure 4.** At 46 days after the first CII immunization, spleens were obtained from vehicle- or daurinol (20 mg/kg, intraperitoneal administration)-treated mice with CIA. **(A)** Flow cytometry was used to identify IL-17+, IFN-γ+, IL-4+, and Foxp3+ cells among CD4+ T cells. **(B)** Flow cytometry was used to identify Treg markers including GITR+, ICOS+, CD103+, CTLA-4+, and PD-1+ cells among CD4+ T cells **(B)**. Representative flow cytometry plots from one of two independent experiments are shown.


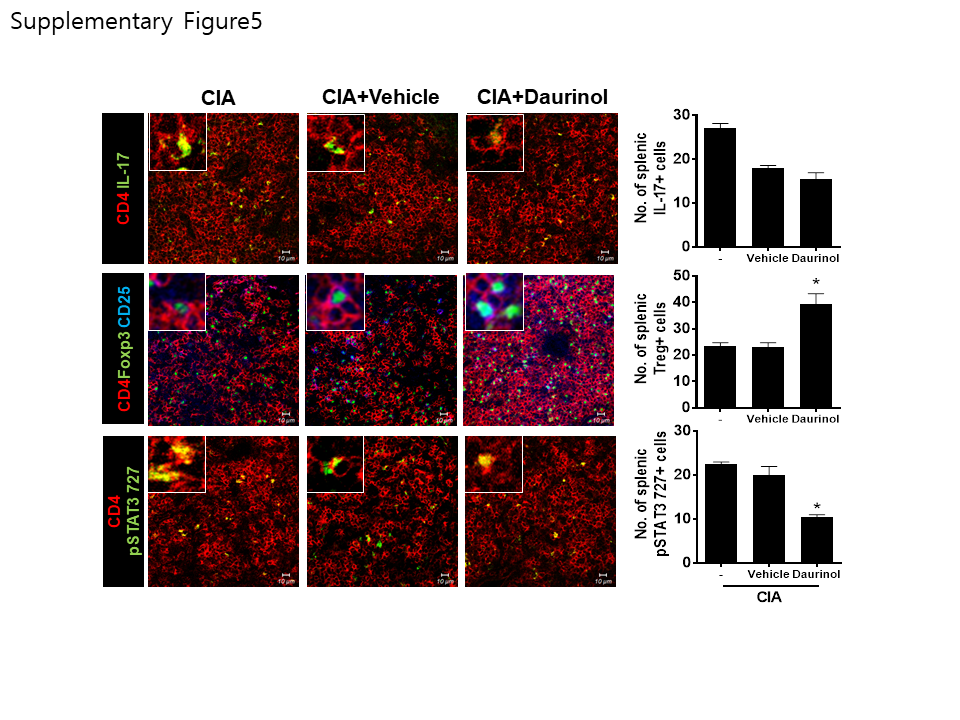
**Supplementary Figure 5.** Spleens were examined by immunofluorescence staining with monoclonal antibodies against CD4 (red), IL-17 (green), Foxp3 (green), CD25 (blue), and pSTAT3 (Ser727) (Original magnification ×40). IL-17+, CD25+Foxp3+, and pSTAT3+ (Ser727) CD4+ T cells were enumerated visually at higher magnification as projected on a screen, with each confocal image representative of four fields of view (scale bar, 10 μM). Values represent the mean ± SD number of positive cells in six mice per group. * *p* < 0.05 versus vehicle-treated CIA mice. Values represent the mean ± SD (bars) results of three independent experiments. ─, untreated


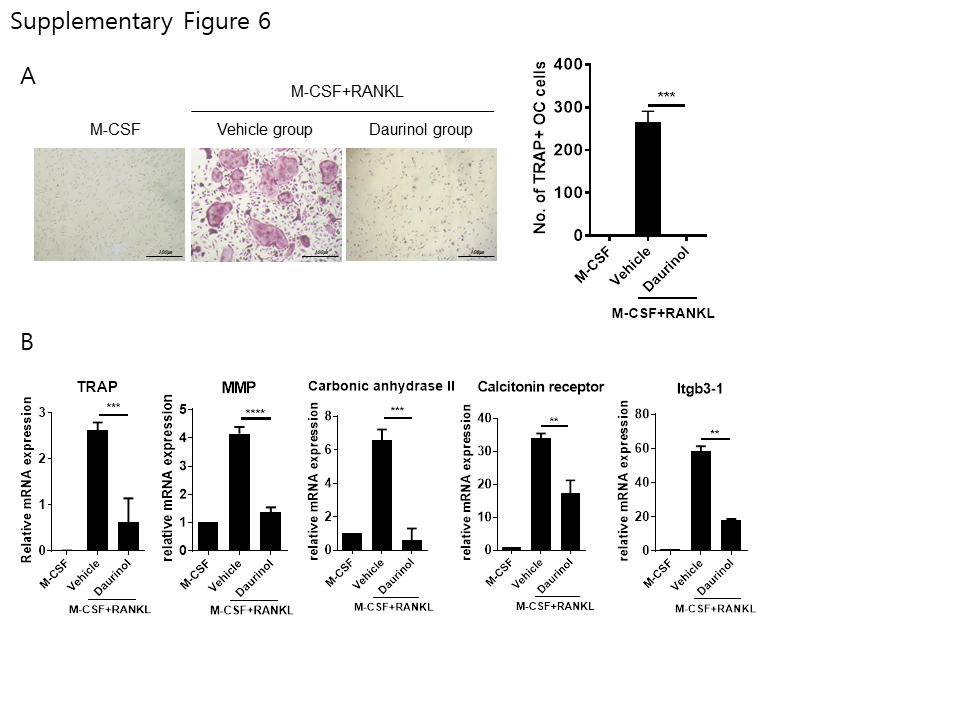


**Supplementary Figure 6. Inhibition of osteoclastogenesis by daurinol. (A)** Bone marrow-derived monocytes/macrophages (BMM) were isolated from wild DBA/1J mice. BMM were cultured for 6 days under conditions for osteoclast differentiation (with 10 ng/ml macrophage colony-stimulating factor [M-CSF] or M-CSF and 50 ng/ml RANKL) in the presence or absence of daurinol (2 μM). The cells were then fixed and stained for tartrate-resistant acid phosphatase (TRAP), and TRAP-positive multinucleated (≥3 nuclei/cell) cells were counted (original magnification ×100, scale bar; 100 μM). **(B)** The relative mRNA levels of osteoclastogenesis-related markers, such as TRAP, MMP9, carbonic anhydrase II, calcitonin receptor, and integrin β3 (*Itgb3*), in BMMs isolated from vehicle- or daurinol-treated CIA mice were evaluated using RT-PCR. Values represent the mean ± SD (bars) for three independent experiments. ** *p* < 0.01, *** *p* < 0.001,

**
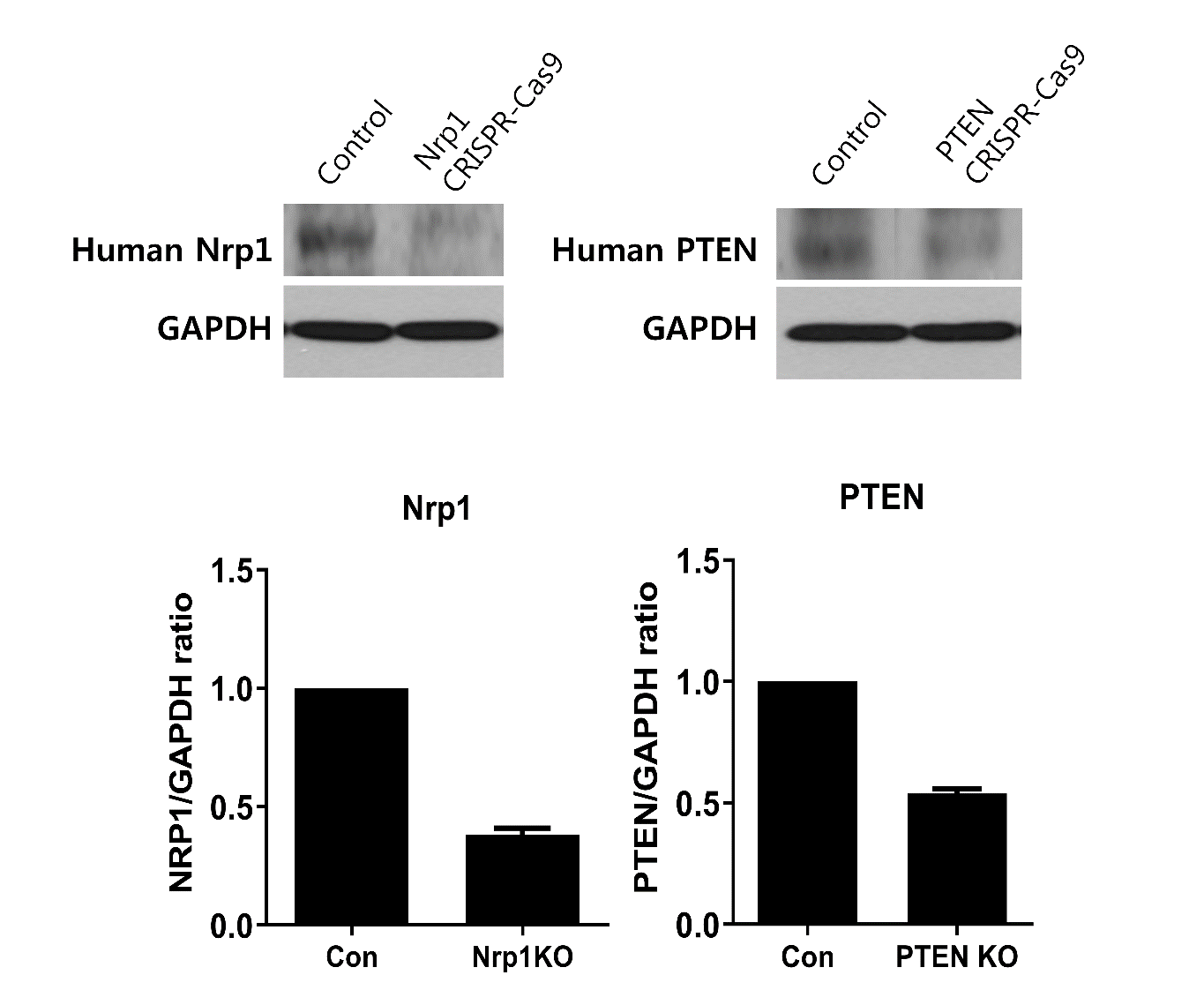
**

**Supplementary Figure7. Nrp1 and PTEN CRISPR knockout in human CD4+ T cells.** Cell lysates were harvested for immunoblotting using antibodies against Nrp1, PTEN and GAPDH as loading control. Data are represented as the mean ± SD (n = 3).

**Table S1. Mouse primers used for real-time PCR**

|  | Forward | Reverse |
| --- | --- | --- |
| IL-17 | CCTCAAAGCTCAGCGTGTCC | GAGCTCACTTTTGCGCCAAG |
| FoxP3 | GGCCCTTCTCCAGGACAGA | GCTGATCATGGCTGGGTTGT |
| RORγt | TGTCCTGGGCTACCCTACTG | GTGCAGGAGTAGGCCACATT |
| Nrp-1 | GACAAATGTGGCGGGACCATA | TGGATTAGCCATTCACACTTCTC |
| HIF-1α | AGCTTCTGTTATGAGGCTCACC | TGACTTGATGTTCATCGTCCTC |
| SOCS3 | CCTTTGACAAGCGGACTCTC | GCCAGCATAAAAACCCTTCA |
| TGFb | CCCTATATTTGGAGCCTGGA | GTTGGTTGTAGAGGGCAAGG |
| Glut1 | CAGTTCGGCTATAACACTGGTG | GCCCCCGACAGAGAAGATG |
| HK2 | TGATCGCCTGCTTATTCACGG | AACCGCCTAGAAATCTCCAGA |
| GPI | TCAAGCTGCGCGAACTTTTTG | GGTTCTTGGAGTAGTCCACCAG |
| TPI | CCAGGAAGTTCTTCGTTGGGG | CAAAGTCGATGTAAGCGGTGG |
| Eno1 | CGCCATGTCTATTCTCAGGATC | AGTTCTAGGGCCTCGTAGATG |
| PKM | GCCGCCTGGACATTGACTC | CCATGAGAGAAATTCAGCCGAG |
| MCT4 | TCACGGGTTTCTCCTACGC | GCCAAAGCGGTTCACACAC |
| KLF2 | CACCTAAAGGCGCATCTGCGTA | GTGACCTGTGTGCTTTCGGTAG |
| IL-10 | GGCCCAGAAATCAAGGAGCA | AGAAATCGATGACAGCGCCT |
| TNF-a | ATGAGCACAGAAAGCATGATC | TACAGGCTGTCACTCGAATT |
| β-actin | GTACGACCAGAGGCATACAGG | GATGACGATATCGCTGCGCTG |

**Table S2. Human primers used for real-time PCR**

|  | Forward | Reverse |
| --- | --- | --- |
| IL-17 | CAACCGATCCACCTCACCTT | GGCACTTTGCCTCCCAGAT |
| FoxP3 | CACTGCCCCTAGTCATGGT | GGAGGAGTGCCTGTAAGTGG |
| Nrp-1 | AAGGTTTCTCAGCAAACTACAGTG | GGGAAGAAGCTGTGATCTGGTC |
| TGFβ | TGCGGCAGCTGTACATTGA | TGGTTGTACAGGGCCAGGA |

**Table S3. Primary antibodies used for Western blot**

| **Target** | **Reactivity** | **Cat No.** | |
| --- | --- | --- | --- |
| Stat3 (124H6) | H M R Mk | Cell signaling | 9139 |
| Phospho-Stat3 (Ser727) | H M R | Cell signaling | 9134 |
| Phospho-Stat3 (Tyr705) (D3A7) | H M R Mk | Cell signaling | 9145 |
| Stat5 (D2O6Y) | H M R | Cell signaling | 94205 |
| Phospho-Stat5 (Tyr694) (C11C5) | H M | Cell signaling | 9359 |
| PTEN | H M R Hm Mk | Cell signaling | 9552 |
| Phospho-PTEN (Ser380) | H M R | Cell signaling | 9551 |
| Total Akt | H, M, R, Mk, Dm, (Pg) | Cell signaling | 4691 |
| pAkt (Ser473) | H, M, R | Cell signaling | 4058 |
| pAkt (Thr308) | H, M,R, Mk | Cell signaling | 13038 |
| Phospho-mTOR (Ser2448) (D9C2) | H M R Mk | Cell signaling | 5536 |
| Neuropilin-1 | H, M, R | Cell signaling | 3725 |
| Smad3 | H, M, R | Cell signaling | 9513 |

**References**

1. Brand DD, Latham KA, Rosloniec EF. Collagen-induced arthritis. *Nat Protoc* (2007) 2:1269-75. doi: 10.1038/nprot.2007.173
